# A novel item-allocation procedure for the three-form planned missing data design

**DOI:** 10.1016/j.mex.2020.100941

**Published:** 2020-05-28

**Authors:** Kyle M. Lang, E. Whitney G. Moore, Elizabeth M. Grandfield

**Affiliations:** a Tilburg University Department of Methodology and Statistics, the Netherlands; b Division of Kinesiology, Health & Sport Studies, Wayne State University, United States; c University of Kansas Medical Center, United States

**Keywords:** Planned missing data, Survey design, Matrix sampling, Questionnaires

## Abstract

We propose a new method of constructing questionnaire forms in the three-form planned missing data design (PMDD). The *random item allocation* (RIA) procedure that we propose promises to dramatically simplify the process of implementing three-form PMDDs without compromising statistical performance. Our method is a stochastic approximation to the currently recommended approach of deterministically spreading a scale's items across the X-, A-, B-, and C-blocks when allocating the items in a three-form design. Direct empirical support for the performance of our method is only available for scales containing at least 12 items, so we also propose a modified approach for use with scales containing fewer than 12 items. We also discuss the limitations of our procedure and several nuances for researchers to consider when implementing three-form PMDDs using our method.

● The RIA procedure allows researchers to implement statistically sound three-form planned missing data designs without the need for expert knowledge or results from prior statistical modeling.

● The RIA procedure can be used to construct both “paper-and-pencil” questionnaires and questionnaires administered through online survey software.

● The RIA procedure is a simple framework to aid in designing three-form PMDDs; implementing the RIA method does not require any specialized software or technical expertise.

## Graphical abstract

**Figure:**
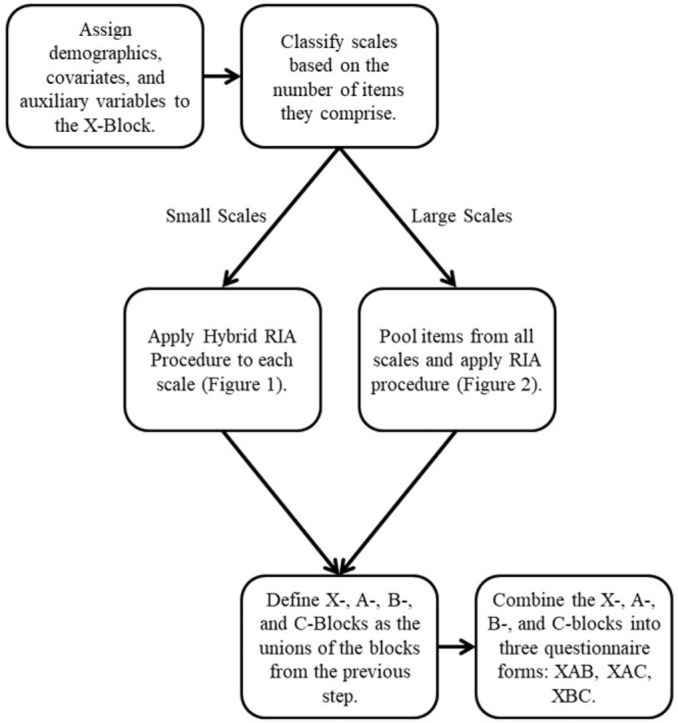

**Table:** Specifications Table

Subject Area:	*Psychology*
More specific subject area:	Psychological Research Methods
Method name:	Random Item Allocation for Three-Form Planned Missing Data Designs
Name and reference of original method	Three-Form Planned Missing Data Design
	Graham, J. W., Hofer, S. M., & MacKinnon, D. P. [4]. Maximizing the usefulness of data obtained with planned missing value patterns: An application of maximum likelihood procedures. *Multivariate Behavioral Research, 31*, 197 – 218.
Resource availability:	NA

## Method details

This article is a companion to Moore et al. [17] and serves two purposes. In the first part of this article, we discuss a novel implementation of the three-form planned missing data design—the random item allocation (RIA) approach—that was shown to perform well in Moore et al. [17]. The RIA approach promises to substantially simplify the process of implementing planned missing data designs, in practice. In the second part, we provide additional details of the methodology of the resampling study reported in Moore et al. [17].

Before proceeding, we provide a brief overview of planned missing data designs (PMDDs) to contextualize the following content. PMDDs are a type of matrix sampling approach wherein researchers intentionally administer incomplete questionnaires to participants. Each participant sees only a subset of the full set of items in the researcher's study. The items that participants do not see become missing values in the final dataset. These missing data are missing completely at random (MCAR) since the researcher defined the missing data patterns a priori (i.e., without consideration for any of the variables in the analysis) and randomly assigned participants to the missing data patterns. Consequently, the planned missing data introduced by a PMDD are easily treated with principled missing data methods like multiple imputation or full information maximum likelihood.

The most common type of PMDD, the three-form design, entails splitting the questionnaire items into four blocks: an X-Block containing items each participant will see and A-, B-, and C-Blocks that contain items only two thirds of the participants will see. After allocating the items to blocks, the researcher creates three questionnaire forms by combining the X-Block items with the items from two of the A-, B-, or C-Blocks. Therefore, in terms of the blocks they comprise, the final set of questionnaires is XAB, XAC, and XBC. For more details on PMDDs, we refer interested readers to Graham [3]; Graham, Hofer, and MacKinnon [4]; Graham, Taylor, Olchowski, and Cumsille [5]; or Little and Rhemtulla [11].

## PMDD item allocation procedures

When researchers implement a PMDD, one of the more difficult decisions they must make is how to allocate items across blocks. This problem has two facets: (1) how to distribute the items between the A-, B-, and C-Blocks, and (2) which items to include in the X-Block. Previous research has suggested that the items within (sub)scales should be divided among the A-, B-, and C-Blocks to maximize covariance coverage between scales [4,7]. The results presented by Moore et al. [17] corroborate the performance of this approach (hereafter the “between-block” assignment method). The natural alternative to the between-block assignment method would be to allocate all the items of a (sub)scale to either the A-, B-, or C-Block. This approach (hereafter the “within-block” assignment method) should not be used when modeling associations among variables because it reduces covariance coverage [7].

Current recommendations suggest that including scale items in the X-Block (in addition to demographic variables) leads to better performance [5], and the results of Moore et al. [17], again, agree. In terms of how to choose the scale items to include in the X-Block, however, current best practice suggests allocating items based on expert knowledge, expected statistical effect sizes, and/or the results of previous modeling [5,11].

The results of Moore et al. [17] suggest a much simpler solution, however. Randomly assigning items to the X-, A-, B-, and C-Blocks does not appear to produce any deleterious effects—at least when the number of items in each scale is reasonably large (i.e., 12 or more items). Moore et al. [17] showed that:

1. Randomly allocating the scale items to the A-, B-, and C-Blocks (without accounting for scale membership) performed just as well as explicitly splitting the items between blocks.
2. Assigning a random subset of the scale items to the X-Block (without accounting for scale membership) performed as well as (or slightly better than) theoretically informed X-Block assignment.

**Fig. 1:**
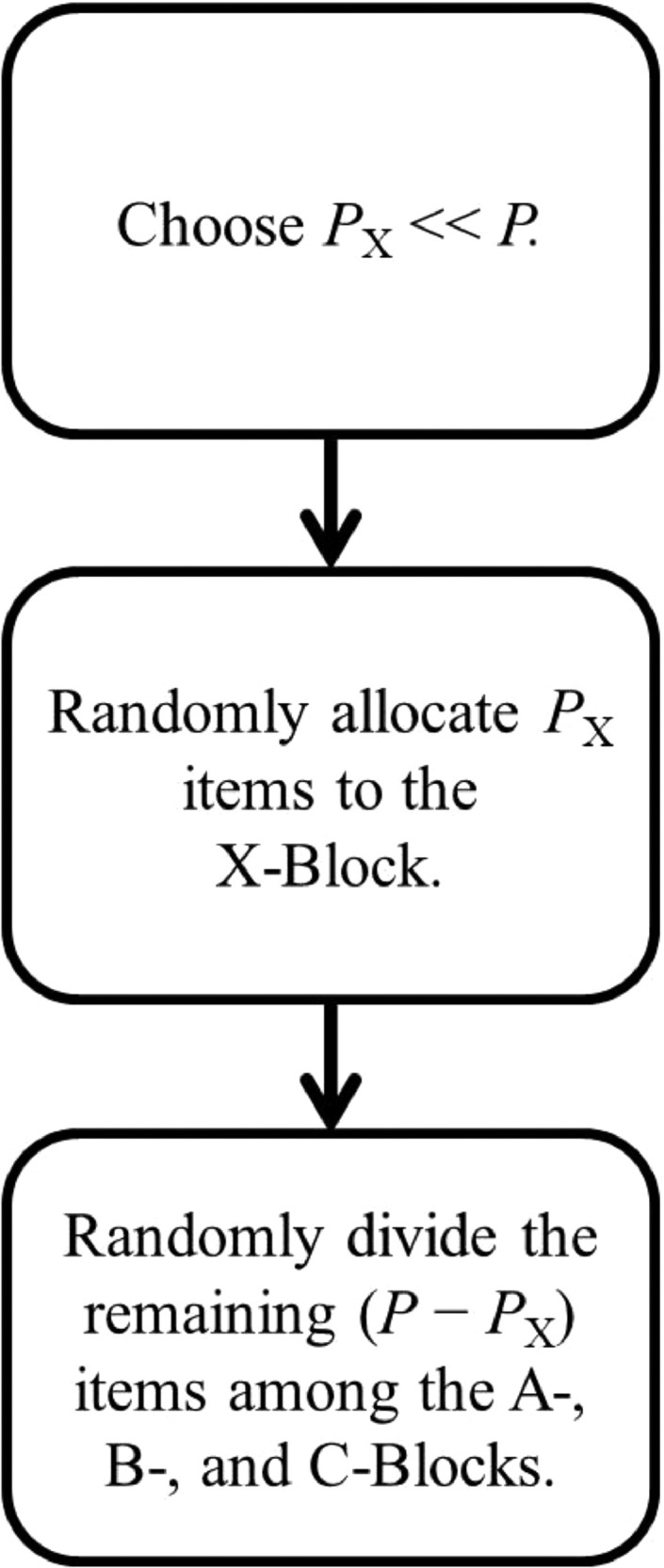
Flowchart describing the logic of the RIA procedure. Note: P = Number of scale items to distribute.

Taken together, these two findings imply that researchers can construct an optimal three-form PMDD by simply deciding how many scale items they wish to include in the X-, A-, B-, and C-Blocks and randomly allocating the scale items to satisfy the desired counts (while assigning all demographics to the X-Block). We call this approach the “random item allocation” (RIA) procedure. Fig. 1 shows a schematic representation of the workflow for distributing scale items among the X-, A-, B-, and C-Blocks using RIA.

In lieu of the three steps shown in Fig. 1, current recommendations dictate first assigning scale items to the X-Block using expert knowledge and/or the results of prior statistical modeling, and then allocating the remaining scale items across the A-, B-, and C-Blocks so that items from the same scale are spread across blocks [7,11]. The RIA procedure does not require expert knowledge, previous results, or explicitly balanced assignment, so RIA substantially simplifies the process of creating and implementing PMDDs.

## Implementation details

Although the RIA procedure appears to work well based on the findings of Moore et al. [17], researchers considering a PMDD should be mindful of certain nuances in the way PMDDs must be implemented with RIA. First, we recommend choosing the number of scale items assigned to the X-Block, *P*_X_, so that the remaining number of items, *P* – *P*_X_, is evenly divisible by three (for the three-form design). Doing so will ensure that the length of each final questionnaire form is equal. Second, although the RIA procedure involves randomly allocating scale items to the X-Block, the X-Block should not necessarily contain only these randomly assigned scale items. Variables in the A-, B-, and C-Blocks will be partially missing in the final dataset, so any items for which missing data is especially undesirable should go into the X-block. A few common examples of such items include:

1. Demographic variables.
2. Important covariates.
3. Auxiliary variables (i.e., covariates that are used for missing data treatment).
4. Any items for which missing data will be especially difficult to address (e.g., outcomes with unusual distributions).

Additionally, it may be worth including any important individual items (e.g., important, univariate predictors or outcomes) in the X-Block. PMDDs work best when they can use strong within-scale associations to support missing data treatment (hence the preference for between-block assignment), and univariate items clearly cannot leverage within-scale associations.

## Caveats, limitations, & extensions

The RIA procedure entails randomly assigning items to blocks, but not every method of randomly allocating items to blocks constitutes an implementation of what we are calling RIA. Many web-based survey programs (e.g., Qualtrics) will generate a novel questionnaire for each participant by randomly sampling from a pool of items. This “on-the-fly” approach to item allocation has been suggested in the literature (e.g., [11]), but we are not aware of any empirical evaluation of its performance. Furthermore, the results of Moore et al. [17] do not directly apply to “on-the-fly” item randomization because the RIA procedure we implemented in this study represents a different type of randomization. For each replication in our study, we generated a new set of (three) questionnaire forms via RIA, but every hypothetical “participant” in our study saw only one of those three forms. The situation modeled in our study, therefore, is one wherein a researcher generates a fixed set of three forms via the RIA procedure and does not update the structure/contents of those forms during data collection (either manually or via the sampling software). The “on-the-fly” item randomization approach is a logical extension of the procedure tested in our study, not an equivalent alternative. Increased computational complexity of the resulting missing data problem is one potential drawback of the “on-the-fly” approach. Randomly generating a, potentially unique, questionnaire form for each participant will increase the number of missing data patterns relative to the three-form design we explore in this study. Although “on-the-fly” randomization will generally produce more missing data patterns, these missing data will still be easily treated MCAR, so we conjecture that the “on-the-fly” approach would perform well, in practice. The veracity of this conjecture is currently under investigation, however, so the results of Moore et al. [17] should not be taken as direct empirical support for “on-the-fly” item randomization.

The RIA procedure is not always an appropriate tool for implementing PMDDs. In certain circumstances, the between-block allocation method is a better way to distribute items to the A-, B-, and C-Blocks. RIA should only be applied to scales that have a relatively large number of items (the number of items required is discussed below). When it comes to allocating items to the A-, B-, and C-Blocks, RIA is a stochastic approximation to the between-block assignment method—RIA works because it tends to split a scale's items across blocks. When applied to scales with few items, the RIA approach will tend to generate solutions wherein some blocks have no items from a given scale while other blocks contain multiple items from the same scale—i.e., solutions that (partially) resemble those produced by the within-block assignment method. In these situations, directly implementing the between-block assignment method is probably the best option. The best approach for a scale comprising only four items, for example, would be to split the four items evenly between the X-, A-, B-, and C-Blocks (i.e., assign one item to each block). Similarly, a scale with fewer than four items should have one item included in the X-Block and the remaining items deterministically distributed between as many of the A-, B-, and C-Blocks as possible. With three items, for example, one item should go into the X-Block, and then one item could go into the A-block and one into the B-Block. The C-Block would not get any items, in this case.

### Hybrid RIA

**Fig. 2:**
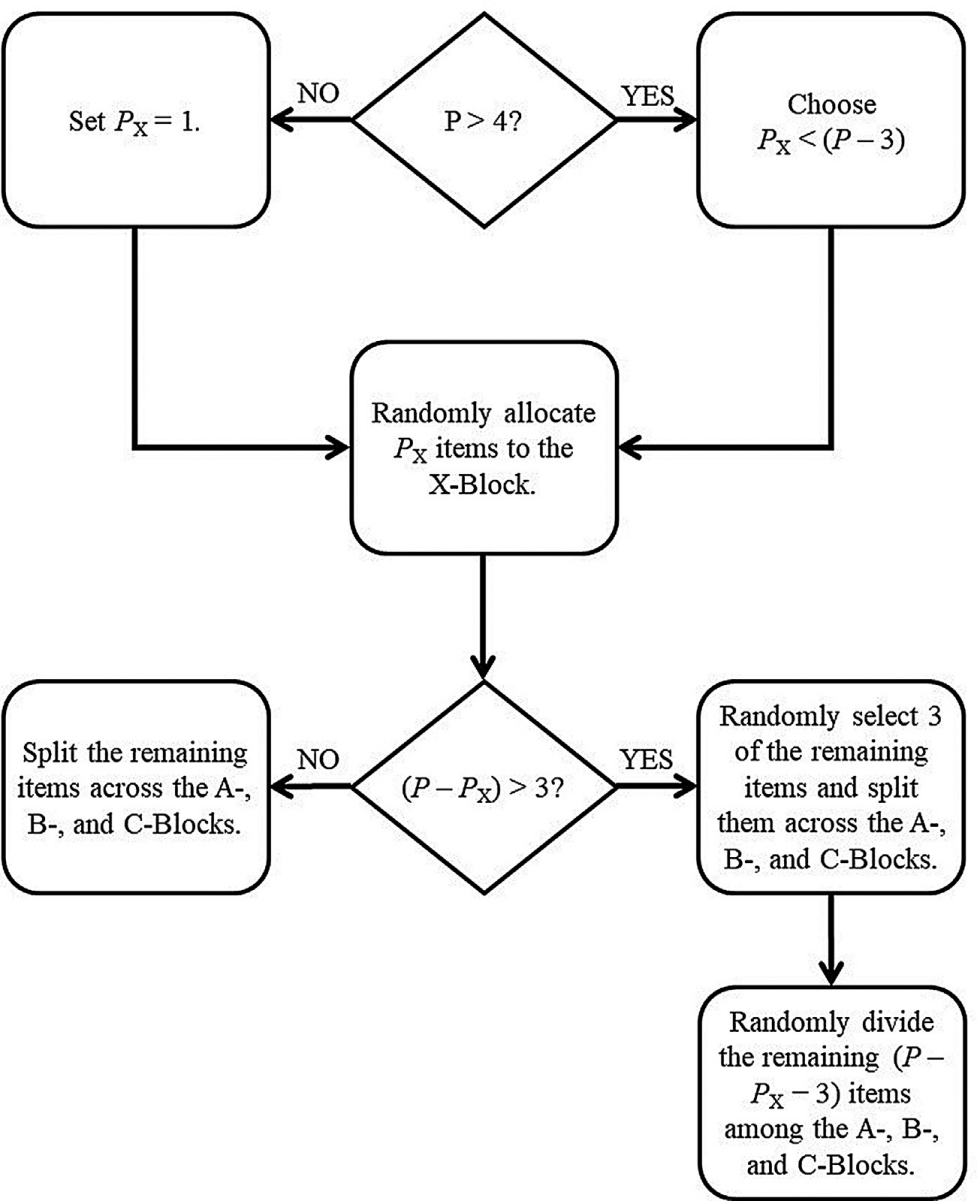
Flowchart describing the logic of the hybrid RIA procedure as applied to a single scale. Note: *P* = Number of items in the scale.

The scales analyzed in Moore et al. [17] contained 13, 13, and 14 items respectively, so the findings suggest that the RIA procedure works well for scales with 13 or more items. That being said, a scale with 12 items would, on average, contribute three items to each block, and a 13th item does not dramatically change the expected item allocation. Therefore, we believe it is reasonable to extrapolate the good performance of the RIA procedure to scales containing 12 or more items. Because the results of Moore et al. [17] do not directly support the use of RIA for scales with fewer than 12 items, we suggest a hybrid approach. For scales that comprise 5 to 11 items, one could use conditional randomization with the requirement that each block must contain at least one item from each scale. Fig. 2 illustrates the workflow for implementing such a hybrid RIA for a scale with few (e.g., less than 12) items. We have not directly evaluated the performance of this hybrid procedure, but we have good reason to expect this approach to perform well. Namely, the hybrid approach combines two item allocations procedures—RIA and between-block assignment—that do have direct empirical support. To implement a PMDD using (hybrid) RIA, we suggest the following procedure:

1. Assign demographics, covariates, auxiliary variables, and other important (or problematic) univariate items to the X-Block (as discussed above).
2. Classify the scales into two groups:
  a. Small Scales (e.g., fewer than 12 items)
  b. Large Scales (e.g., 12 or more items)
3. Pool the items from all large scales and make X-, A-, B-, and C-Blocks by following the RIA logic outlined in Fig. 1.
4. For any small scales, make X-, A-, B-, and C-Blocks by following the hybrid RIA logic outlined in Fig. 2.
5. The final X-, A-, B-, and C-Blocks are the union of the X-, A-, B-, and C-Blocks created in Steps 3 and 4.
6. Combine the final X-, A-, B-, and C-Blocks into the three questionnaire forms (i.e., XAB, XAC, XBC).

Any univariate items that are not important enough to include in the X-Block can be randomly allocated among the A-, B-, and C-Blocks. This procedure is represented graphically in the visual abstract for this paper.

Practically speaking, researchers can implement the random assignment described above by using a random number generator like those available in Excel, SPSS, SAS, or R (as well as numerous other sources, including mobile and web-based applications). The procedure outlined above will produce a one-time random assignment of items to the X-, A-, B-, and C-Blocks. These blocks can then be combined into three printed questionnaire forms (i.e., XAB, XAC, and XBC). Alternatively, researchers can define the blocks in online survey software and set the sampling routine to randomly present two of the A-, B-, and C-Blocks to each study participant after they complete the X-block. Either approach will result in randomly presenting one of the three questionnaire forms to each participant. The “on-the-fly” approach, on the other hand, could potentially present a different combination of items to each participant.

When applying the basic RIA procedure, there is a possibility that some scales may not have any items assigned to the X-block. The risk of this issue is greater for smaller scales. The hybrid RIA procedure avoids this possibility, so it may be desirable to apply the hybrid RIA procedure even when the number of scale items is sufficient to justify basic RIA (i.e., 12 or more). Hybrid RIA is not without its limitations, however. Implementing hybrid RIA is more complicated than implementing basic RIA. So, using hybrid RIA, in lieu of basic RIA, entails more effort and increases the chances for implementation errors. Researchers interested in a pragmatic alternative to hybrid RIA that avoids the above issues could simply apply basic RIA and check the resulting X-block allocation. If any scales are not represented in the X-block, the researcher can rerun the basic RIA procedure until an acceptable X-block is generated.

### Extended methods of the resampling study

In this section, we provide additional methodological details of the resampling study reported in Moore et al. [17]. We conducted this resampling study to evaluate the performance of different instantiations of the three-form PMDD in an ecologically valid fashion. The original data from which we sampled (hereafter, the “population data”) were collected by Moore and Fry [15] to study the effects of motivational climate perceptions on exercise participants’ class ownership and enjoyment. We excluded cases from the population data that met either of the following criteria: (1) had a missing *race* value or (2) endorsed a *race* category that represented less than 1% of the sample size. We implemented these exclusion criteria for four reasons:

1. Imputing/analyzing nominal variables was not the focus of our study.
2. Nominal variables are notoriously difficult to impute [9].
3. Sparse categorical variables often cause estimation problems [1]
4. Nominal variable imputation tends to be very slow, so retaining missing *race* values would substantially extend the computation time of our study without adding any scientific benefit.

The resulting population data contained *N* = 5244 participants of which 98.5% self-identified as female (0.65% missing) and 90.2% self-identified as white. The average observed participant age was 49.27 years (SD = 11.09, 1.47% missing). All variables except *race* had a small amount of missing data. The variable-wise percentages of missing data ranged from 0.04% to 1.47%. For further details of the population data collection and characteristics see Moore and Fry [15].

### Variables

In the population data for this study, we included three of the original five constructs collected by Moore and Fry [15]. Specifically, 13 items assessing ego-involving climate and 14 items assessing task-involving climate from the Perceived Motivational Climate in Exercise Questionnaire (PMCEQ; [6]), and 13 items from the Caring Climate Scale (CCS; [19]). For more information about the PMCEQ or CCS, see Moore et al. [17] or Moore and Fry [15]. We also included indicators of participant age, biological sex, and race.

### Resampling

For each replication of the resampling study, we drew a random sample (with replacement) of size *N* = 500 from the population data described above. Rather than draw new samples for the *N* ∈ {400, 300, 200, 100} conditions, we recursively “trimmed” observations from the original sample of *N* = 500. For the results reported in Moore et al. [17], we retained all extant missing data during the resampling processes. When we ran the study using only complete cases as the population data, the results were essentially equivalent to those derived from the incomplete population data.

## Imposing planned missing data

Within each resampled (or trimmed) dataset, we imposed planned missing data according to nine different instantiations of the three-form design. These versions differed in terms of two crossed factors: the composition of the X-Block and the way in which we assigned items to the A-, B-, and C-Blocks. The *X-Block* factor had three levels:

1. A trivial X-Block that contained only sex, age, and race.
2. An informed X-Block that contained the demographic variables listed in (1) and items chosen with guidance from previous CFA models [6,14].
3. A random X-Block that contained the demographic variables listed in (1) and randomly selected scale items.

See Moore [13] and Moore and Fry [16] for more information regarding the development of the informed X-Block and the parceling scheme.

The *Parcel* factor also contained three levels:

1. A within-block condition wherein we assigned all items of each parcel to either the A-, B-, or C-Block.
2. A between-block condition wherein we distributed the items of each parcel across the A-, B-, and C-Blocks.
3. A random-allotment condition wherein we randomized the assignment of items to the A-, B-, and C-Blocks.

In the random X-Block and the random parcel conditions, we generated a new random assignment for every replication of the resampling study. The combination of the random X-Block and random-allotment methods constitutes the RIA approach discussed in the first part of this article.

## Analysis model

The analysis model from which we derived the parameter estimates used to evaluate the different versions of PMDD was a confirmatory factor analysis (CFA) with standardized latent variables (i.e., the measurement scale was set with the so-called “fixed factor” method of identification). The latent correlation structure was fully saturated, and all item intercepts, factor loadings, and residual variances were freely estimated. Each latent factor loaded onto three parceled indicators. We calculated the parcel scores after imputing the data (i.e., a unique set of parcels was computed from each of the *M* = 100 imputed datasets). To evaluate the relative performance of the different PMDDs, we considered the effects on latent correlations, factor loadings, item intercepts, and residual variances.

## Outcome measures

To evaluate the relative performance of the different implementations of PMDD, we compared latent reliabilities as well as biases and efficiencies of the parameter estimates noted above.

### Latent reliability

Following Bollen [2] and Raykov [21], we define latent reliability as:

$$\rho \left(Y_{j}\right)=\frac{{\left(\sum_{i=1}^{I}\lambda_{ij}\right)}^{2}\psi_{jj}}{{\left(\sum_{i=1}^{I}\lambda_{ij}\right)}^{2}\psi_{jj}+\sum_{i=1}^{I}\theta_{ii}}$$

where *Y_j_* is the scale score (i.e., sum of the observed items) for the *j*th scale, *λ_ij_* is the factor loading linking the *i*th indicator to the *j*th latent construct, *ψ_jj_* is the latent variance for the *j*th construct, and *θ_ii_* is the residual variance for the *i*th indicator. Latent reliability, similar to Cronbach's alpha coefficient, can be viewed as the squared correlation between an observed scale score (i.e., the sum of the item scores) and that scale's true score [2,21]. Unlike Cronbach's alpha, however, the quantities that go into computing latent reliability are derived from a latent variable model, so they are not contaminated by measurement error. As with Cronbach's alpha, *ρ*(*Y*) is bounded by 0.0 and 1.0 (higher values indicate greater reliability).

### Relative efficiency (RE)

We calculated the RE of each estimated parameter (i.e., latent correlations, factor loadings, item intercepts, and residual variances). RE is defined as:

$$RE=R^{-1}\sum_{r=1}^{R}\frac{SE{\left(\theta \right)}_{r}}{SE{\left(\hat{\theta }\right)}_{r}}$$

where *SE*(*θ*)_*r*_ is the standard error for the parameter in the complete data control condition (i.e., the condition wherein we did not impose any planned missing data), $SE{\left(\hat{\theta }\right)}_{r}$ is the standard error for the parameter in the planned missing condition, and *r = 1, 2, …, R* indexes replication of the resampling study. In our study, RE quantifies the loss of efficiency (i.e., the increase in sampling variability) introduced by the planned missing data (relative to data with only naturally occurring missing data). A value of RE = 1.0 would indicate no loss of efficiency; whereas a value of RE < 1.0 indicates some loss of efficiency (smaller values indicate greater losses).

### Percent relative bias (PRB)

We also calculated the PRB for each estimated parameter and latent reliability. PRB is defined as:

$$PRB=100\left(\frac{\bar{\hat{\theta }}-\theta }{\theta }\right)$$

where $\bar{\hat{\theta }}=R^{-1}\sum_{r=1}^{R}{\hat{\theta }}_{r}$ is the average of the estimated parameters and *θ* is the true value of the parameter. In this study, we took the averages of the complete data parameter estimates (i.e., those estimates derived from data with no planned missing) as the “true” parameter values. PRB gives a measure of bias (i.e., the expected difference between the estimated and true parameters) as a percentage of the true parameter value. Absolute values of PRB larger than 10 are often viewed as indicative of “unacceptable” levels of bias [18].

### Convergence failures

In addition to evaluating bias and efficiency, we also tracked four types of convergence failure:

1. Complete failures of an entire study replication (i.e., runs wherein the program crashed for an indeterminate reason).
2. Failures of the imputation process (i.e., fatal errors returned by the program when imputing the missing data).
3. Non-convergent CFA models (i.e., runs wherein either the program crashed when estimating the CFA models or the maximum likelihood estimator of the CFA models did not converge).
4. CFA models that converged to inadmissible solutions (i.e., Heywood cases)

## Software & computing environment

We conducted all analyses using the R statistical programming language [20]. To treat the missing data (both planned and un-planned), we used the *mice* package [27] to generate 100 imputed datasets using 20 iterations of the chained equations algorithm. Before running the full resampling study, we conducted a small number of test runs wherein we checked the convergence of the imputation models by examining trace plots of the imputed values’ means and standard deviations. We used predictive mean matching [10,23] as the elementary imputation method because it tends to perform well with non-normally distributed, quasi-continuous items such as those in our data [26].

We estimated the CFA models using ordinary maximum likelihood estimation in the *lavaan* package [22]. We pooled the multiply imputed parameter estimates using the Rubin [24] pooling rules as implemented in the *mitools* package [12]. The online supplementary material includes the R scripts used for this study.

The resampling study was run in parallel on the Lisa high performance computing cluster (https://www.surf.nl/en/lisa-compute-cluster-extra-processing-power-for-research) that is administered by SURFsara (https://www.surf.nl/en). We used the routines in the *parallel* [20] package to parallelize the computations of our study across nodes of the Lisa cluster. We used the message passing interface (MPI) protocol provided by the *parallel* package to implement the parallelization. All pseudorandom numbers were generated with the L'ecuyer, Simard, Chen, and Kelton [8] method as implemented in the *rlecuyer* package [25].

## Procedure

Our final design comprised 3(*X-Block*) × 3(*Parcel*) × 5(*Sample Size*) = 45 fully crossed conditions. Within each condition, we ran *R* = 495 replications. As noted above, each replication began by randomly sampling *N* = 500 observations from the population data. To generate samples with *N* < 500, we “trimmed down” the current working dataset by removing 100 observations. We repeated this process, recursively, to create samples with *N* ∈ {400, 300, 200, 100}. At each level of *N*—before imposing the planned missing data—we fit the analysis model to the full data and saved the parameter estimates for the complete data control condition that would define the “true” population values (as described above).

## Supplementary material and/or Additional information

A ZIP archive containing the R scripts used to conduct this resampling study is available as online supplementary material.

## Declaration of Competing Interest

The authors declare that they have no known competing financial interests or personal relationships that could have appeared to influence the work reported in this paper.

## Appendix Supplementary materials

Image, application 1
